# Aging impairs the ability of vascular endothelial stem cells to generate endothelial cells in mice

**DOI:** 10.1007/s10456-023-09891-8

**Published:** 2023-08-10

**Authors:** Shota Shimizu, Tomohiro Iba, Hisamichi Naito, Fitriana Nur Rahmawati, Hirotaka Konishi, Weizhen Jia, Fumitaka Muramatsu, Nobuyuki Takakura

**Affiliations:** 1 Department of Signal Transduction, Research Institute for Microbial Diseases, Osaka University, 3-1 Yamada-oka, Suita, Osaka, 565-0871 Japan; 2 Department of Anatomy, Keio University School of Medicine, Tokyo, Japan; 3 Department of Physiology, Kanazawa University School of Medicine, Ishikawa, Japan; 4 World Premier Institute Immunology Frontier Research Center, Osaka University, Osaka, Japan; 5 Integrated Frontier Research for Medical Science Division, Institute for Open and Transdisciplinary Research Initiatives (OTRI), Osaka University, Osaka, Japan; 6 Center for Infectious Disease Education and Research, Osaka University, Osaka, Japan

**Keywords:** Endothelial cells, Aging, Stem cell aging, Vascular regeneration, Inflammation

## Abstract

Tissue-resident vascular endothelial stem cells (VESCs), marked by expression of CD157, possess long-term repopulating potential and contribute to vascular regeneration and homeostasis in mice. Stem cell exhaustion is regarded as one of the hallmarks of aging and is being extensively studied in several types of tissue-resident stem cells; however, how aging affects VESCs has not been clarified yet. In the present study, we isolated VESCs from young and aged mice to compare their potential to differentiate into endothelial cells in vitro and in vivo. Here, we report that the number of liver endothelial cells (ECs) including VESCs was lower in aged (27–28 month-old) than young (2–3 month-old) mice. In vitro culture of primary VESCs revealed that the potential to generate ECs is impaired in aged VESCs isolated from liver and lung relative to young VESCs. Orthotopic transplantation of VESCs showed that aged VESCs and their progeny expand less efficiently than their young counterparts when transplanted into aged mice, but they are equally functional in young recipients. Gene expression analysis indicated that inflammatory signaling was more activated in aged ECs including VESCs. Using single-cell RNA sequencing data from the Tabula Muris Consortium, we show that T cells and monocyte/macrophage lineage cells including Kupffer cells are enriched in the aged liver. These immune cells produce IL-1β and several chemokines, suggesting the possible involvement of age-associated inflammation in the functional decline of VESCs with age.

**Supplementary Information:**

The online version contains supplementary material available at 10.1007/s10456-023-09891-8.

## Introduction

Endothelial cells (ECs) line the lumen of blood vessels and play essential roles in homeostatic vascular functions such as the regulation of vascular permeability, vasodilator responses, angiogenesis and the secretion of angiocrine factors [1]. In adult individuals, ECs are quiescent in their steady state but still retain proliferative potential in response to angiogenic stimuli such as vascular endothelial growth factor (VEGF). Blood vessel formation from pre-existing vessels, known as angiogenesis, is triggered by hypoxia or inflammation when neovascularization is required [2]. ECs transiently commit to the specific cell fates known as tip or stalk cells during angiogenesis [3]. Tip cells appear at the leading front of angiogenic vessels and guide endothelial sprouting, followed by highly proliferative stalk cells. This tip-stalk selection is strictly controlled by VEGF and Notch signaling and changes dynamically depending on the signal strength [4]. These studies on mouse retinal angiogenesis suggested that this fate commitment is simply stochastic, initiated by the VEGF gradient and the availability of Notch receptors and their ligands.

However, several recent reports documented clonal expansion of ECs in adult mice, indicating the presence of a highly proliferative subpopulation of ECs playing pivotal roles during angiogenesis [5, 6]. Such highly proliferative EC progenitors were first isolated from circulating blood and designated circulating endothelial progenitor cells (EPCs) [7]. Apart from these circulating EPCs, endothelial progenitors with colony-forming potential were also found in the vessel walls [8]. Recently, several groups including ourselves have reported the presence of tissue-resident vascular endothelial stem cells (VESCs) in the peripheral vessel walls, and have proposed specific markers for their identification [9–15]. We have previously reported that CD157 (also known as Bst1) is a specific marker for VESCs possessing colony-forming ability in vitro and long-term repopulating potential in vivo, thus contributing to the entire vasculature of the liver [14].

Stem cell exhaustion in somatic organs is regarded as one of the hallmarks of aging [16]. Age-dependent changes in stem cells have been extensively studied in several types of tissue-resident stem cells [17–21]. For instance, hematopoietic stem cells (HSCs) increase their number during aging, while the differentiation potential of each individual HSC is impaired [17]. Conversely, muscle stem cells (satellite cells), as well as melanocyte stem cells, are decreased both in number and proliferative potential with age [19, 21]. These studies on stem cell aging indicate that age-dependent alterations in stem cells are cell type-specific or context-dependent. Although these findings highlighted the importance of research focusing on a particular type of stem cells, how aging affects VESCs has yet to be elucidated.

In the present study, we explored age-dependent differences of VESCs, mainly focusing on their number, potential to generate ECs, and their gene expression signatures. We have previously reported that upon deletion of TGF-b-activated kinase-1 (TAK1), liver ECs are highly sensitive to tumor necrosis factor-α (TNF-α)-induced apoptosis during inflammation [22]. Because a pro-inflammatory state is one of the best-known features of aging [16], we mainly focused on VESCs in the liver in the present study. We characterized VESCs as well as the whole population of ECs in the mouse liver from younger adult (2–3 month-old) and aged (27–28 month-old) mice. We also examined the impact of the microenvironment in aged mouse liver by transplantation of VESCs from aged mice to young mice. Furthermore, we evaluated age-associated gene signatures by analyzing gene expression patterns of liver ECs using RNA sequencing (RNA-seq). We also utilized single-cell RNA-seq (scRNA-seq) data from the Tabula Muris Senis Consortium to determine immune cell profiles of aged livers. We report a possible involvement of aging-induced inflammation, so-called inflammaging, in the functional impairment of VESCs with age.

**Fig. 1:**
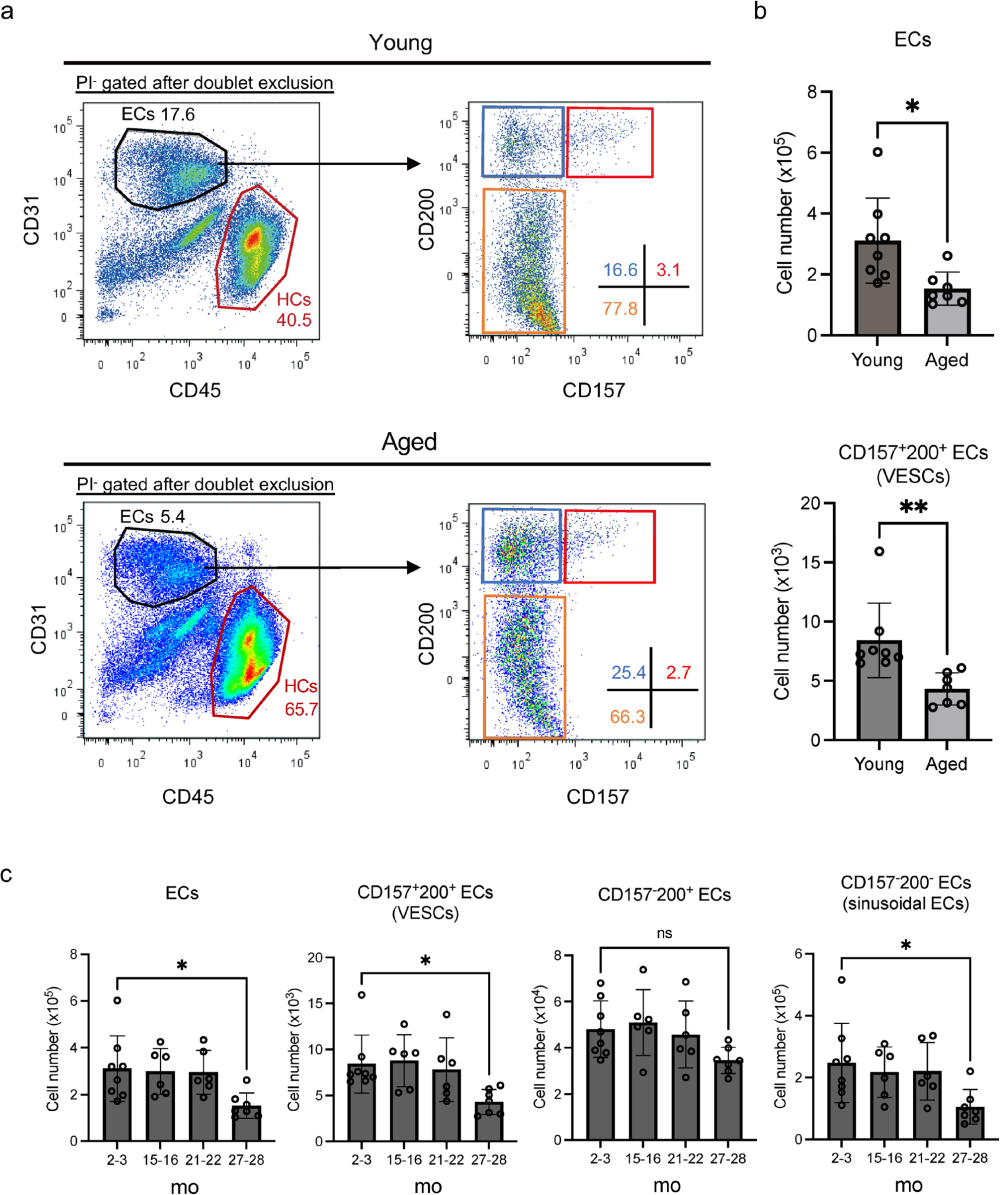
Liver ECs including VESCs decrease with age. **a** Flow cytometry of liver ECs from young (2–3 month-old) and aged (27–28 month-old) mice. Representative FACS plots of young and aged livers are shown in the upper and lower panels, respectively. Each left-hand plot shows the PI^−^-gated population after doublet exclusion. CD31^+^CD45^−^ ECs are further analyzed for their CD157 and CD200 expression in the right-hand panels. Numerals shown in the plots indicate the proportion of the indicated cell population within each plot. HCs: hematopoietic cells (CD31^−^CD45^+^). **b** Quantification of ECs (CD31^+^CD45^−^; upper) and CD157^+^200^+^ VESCs (lower) in the whole liver (*n* = 7). Statistical significance was assessed with two-tailed unpaired Student’s *t* tests. **c** Quantification of total ECs, CD157^+^200^+^ ECs, CD157^−^200^+^ ECs and CD157^−^200^−^ ECs in the whole liver from 2–3, 15–16, 21–22 and 27–28 month-old (mo) mice (*n* = 6–8). CD157^−^200^−^ ECs represent sinusoidal ECs. Statistical significance was evaluated with one-way analysis of variance (ANOVA) followed by Tukey’s multiple comparison test. *P < 0.05, **P < 0.01 or *ns*  not significant

## Results

### Decline of liver ECs with aging coincides with the loss of VESCs

**Fig. 2:**
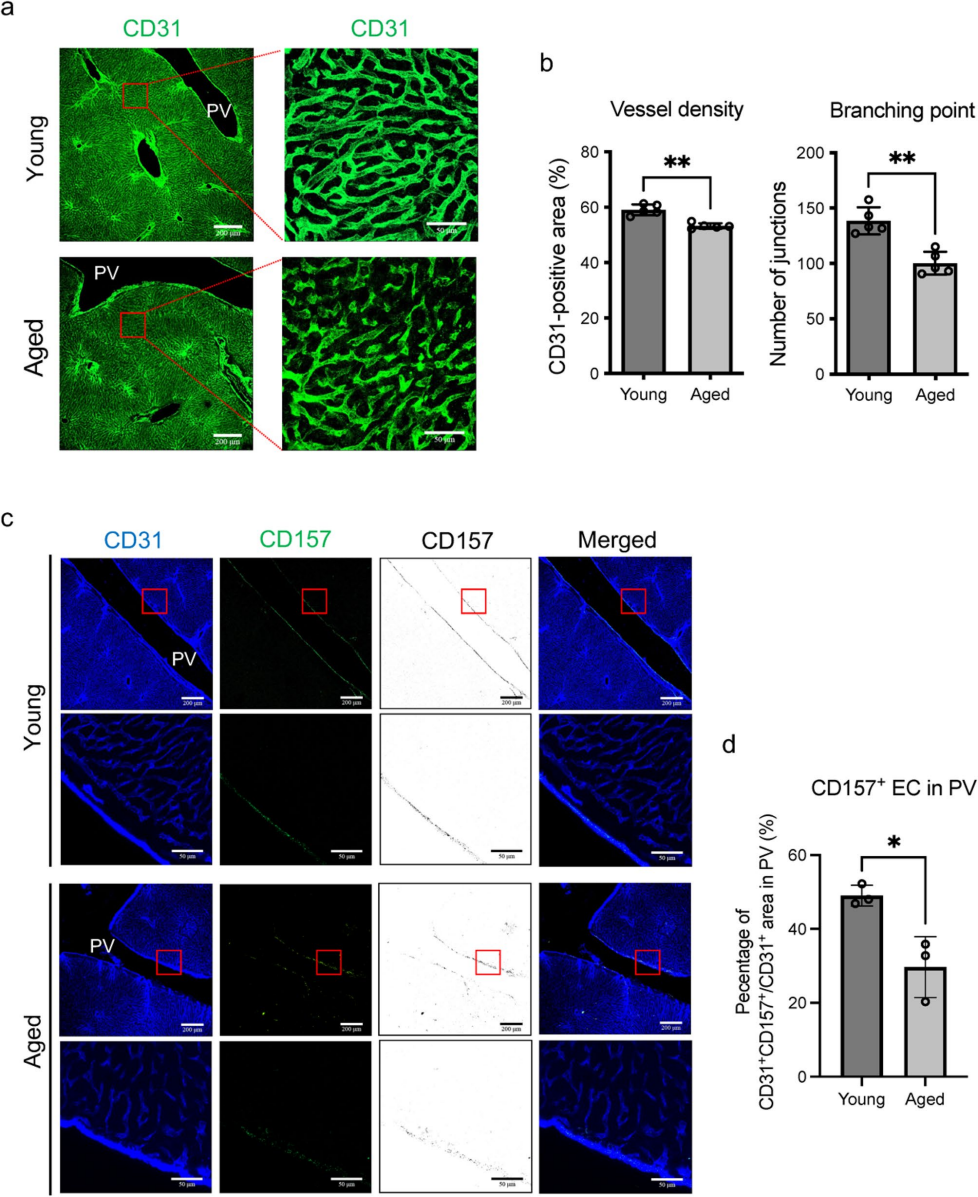
Disorganization of liver sinusoidal vessels coincides with the loss of CD157^+^ ECs in the portal veins with age.** a** Immunohistochemical staining of young (2-3 month-old) and aged (28-month-old) livers with anti-CD31 antibody (green). Higher magnifications of the areas indicated by the red box in the left-hand panels are shown in the right-hand panels. Scale bars represent 200 μm (left) and 50 μm (right), respectively. **b** Quantification of vessel density and branching points of sinusoidal vessels in (**a**). (*n* = 5). **c** Immunofluorescence staining of young and aged livers with anti-CD31 (blue) and anti-CD157 (green, black) antibody. The areas indicated by the red boxes in the first and third panels from the top are magnified in the images below each panel. CD157^+^ ECs predominantly localize to the portal vein (PV) in both young and aged liver. Scale bars denote 200 μm (low magnification) and 50 μm (high magnification). **d** Quantification of CD157^+^ ECs at the PV in (**c**) (*n* = 3). Abundance of CD157^+^ ECs is expressed as the proportion of CD31^+^CD157^+^ areas over the total CD31^+^ areas in the PV. Statistical significance was assessed with two-tailed unpaired Student’s *t* tests in (**b**) and (**d**). *P < 0.05 or **P < 0.01.

First, we compared the number of liver ECs isolated from young (2–3 month-old) and aged (27–28 month-old) mice using fluorescence-activated cell sorting (FACS). Body, liver and lung weights all tended to be higher in 15–16 month-old mice than in young mice and to remain almost the same in mice older than 15 months (Fig. S1a). The ratio of body to liver weight was mostly constant among age groups. Serum alanine aminotransferase (ALT) levels did not significantly differ among age groups (Fig. S1b). These data suggest that there was no overt malignancy or severe liver injury in the aged mice. Liver ECs can be divided into 3 fractions based on their expression of CD157 and CD200 antigens. CD157^+^200^+^ ECs represent the VESC population and mainly localize to the intraluminal surface of portal veins. CD157^−^200^−^ ECs are mature sinusoidal ECs with low proliferative potential. CD157^−^200^+^ ECs, which possess proliferative ability intermediate between CD157^+^200^+^ and CD157^−^200^−^ ECs, reside in large vessels such as portal veins, hepatic veins and hepatic arteries [14]. The number of CD157^+^200^+^ VESCs as well as all ECs (CD31^+^CD45^−^) was significantly lower in aged than young mice (Fig. 1a and b). In contrast, the hematopoietic cell population (CD31^−^45^+^) was enriched in aged mice (young vs. adult, 40.5 ± 7.8% vs. 65.7 ± 10.7% of total cells, *p* < 0.01, *n* = 7). We quantified liver ECs at several ages and found that their numbers were not lower in mice 21–22 months of age but were markedly lower in 27–28 month-old animals (Fig. 1c). Although there were significantly fewer CD157^+^200^+^ and CD157^−^200^−^ ECs at 27–28 months of age, the loss of CD157^−^200^+^ ECs was relatively modest and did not achieve statistical significance. Next, we analyzed the liver vasculature by immunofluorescence. Liver sinusoidal vessels were disorganized in aged mice, characterized by decreased vascular density and branching points (Fig. 2a and b). CD157^+^200^+^ ECs predominantly localized at the portal vein in both aged and young mice, but less frequently expressed CD157 in old animals (Fig. 2c and d). CD157-negative ECs in the portal veins of aged mice were still CD200-positive (Fig. S2a). Taken together with the data from FACS analysis, we conclude that the CD157^+^200^+^ VESC population and CD157^−^200^−^ sinusoidal EC population are decreased with age at their originally localized area/region.

### Aging impedes VESC endothelial colony formation in vitro

**Fig. 3:**
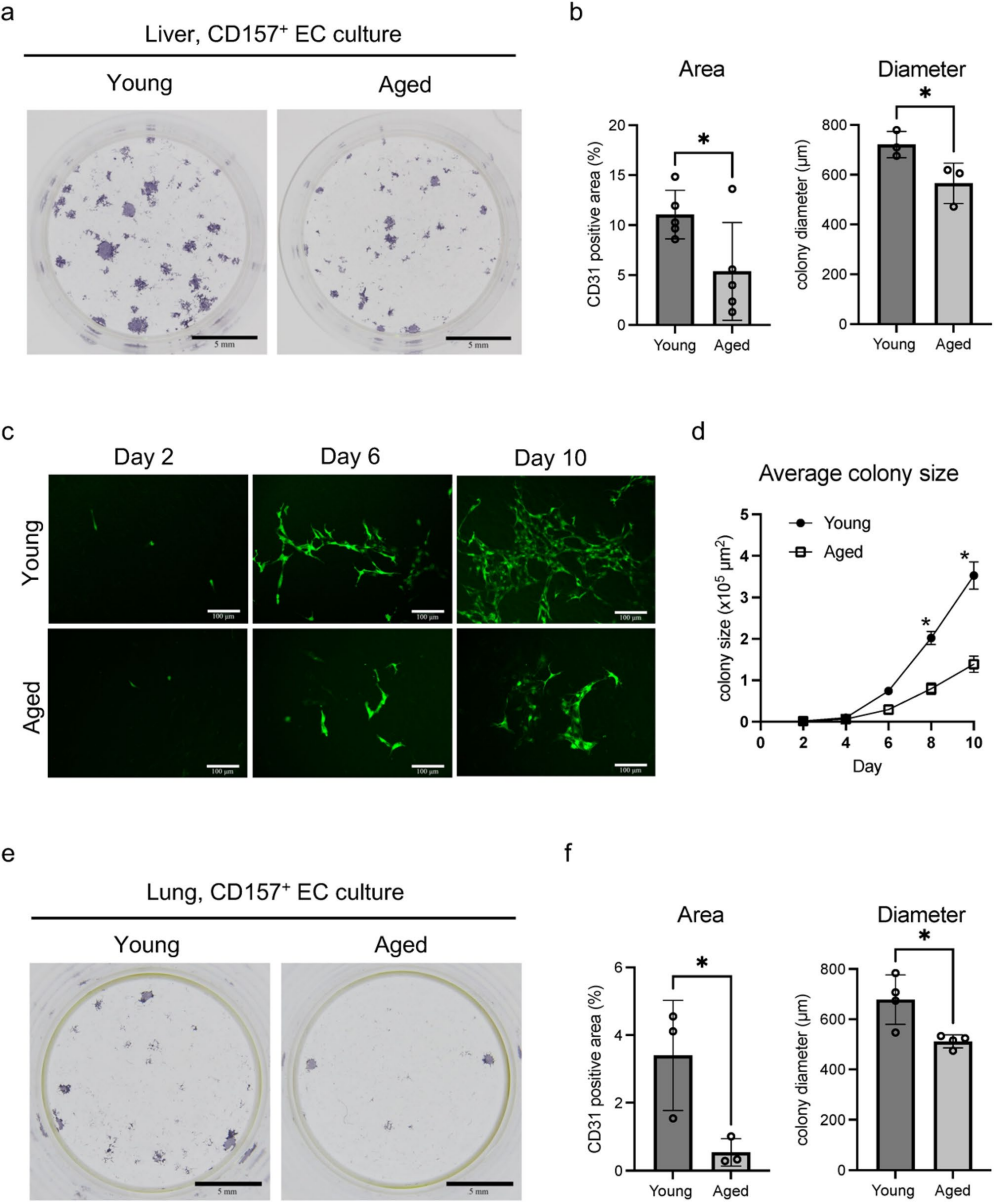
Colony-forming potential of VESCs is impaired with age. **a** Colony formation of liver VESCs on OP9 feeder cells. One thousand CD157^+^CD200^+^ VESCs isolated from young (2–3 month-old) and aged (26 month-old) mouse livers were cultured on OP9 cells for 10 days, followed by immunostaining with anti-CD31 antibody. Scale bars represent 5 mm. **b** Quantification of CD31-positive colony area and average colony diameter in (**a**) (*n* = 5). **c** Time course of colony formation analyzed with EGFP^+^ VESCs. One thousand VESCs isolated from young and aged EGFP mouse liver were cultured on OP9 cells. Fluorescence images of each EGFP^+^ colony were captured at the same position of the culture plate at day 2, 4, 6, 8 and 10 after seeding. Representative images of EGFP^+^ colonies at day 2, 6 and 10 are shown. Scale bars denote 100 μm. **d** Quantification of the average EGFP^+^ colony size at the observed time points in (**c**) (*n* = 3). **e** Colony formation assays of lung VESCs. Five thousand CD157^+^CD200^+^ VESCs isolated from young and aged mouse lung were cultured on OP9 feeder cells for 10 days, followed by immunostaining using anti-CD31 antibody. Scale bars represent 5 mm. **f** Quantification of total colony area and average colony diameter in (**e**) (*n* = 3). Statistical significance was assessed with two-tailed unpaired Student’s *t* tests in (**b**) and (**f**). Two-way ANOVA was used in (**d**). *P < 0.05.

We next investigated functional changes of VESCs with age. First, we performed colony-forming assays using CD157^+^200^+^ VESCs isolated from young and aged mice and found that VESCs sorted from the liver of aged mice formed less colonies than from young mice (Figs. 3a, b and S2b). The average size of the colonies derived from aged VESCs was also smaller, indicating that the EC-producing ability of individual VESCs is decreased with age. This finding was further confirmed by colony-forming assays using ECs isolated from EGFP mice, which helped us to observe the process of colony formation. We observed colonies at the same place and found that aged VESC-derived colonies grow more slowly. This difference between young and aged VESCs was statistically significant 8 days after seeding (Fig. 3c and d). We additionally analyzed the colony-forming ability of CD157^+^ VESCs from the lung, which was also blunted in aged mice, similar to the liver VESCs (Fig. 3e and f). These results show that the ability of VESCs to generate ECs is disrupted during aging, at least in vitro.

### Aged VESCs expand less efficiently than young VESCs when transplanted into aged but not into young recipients

**Fig. 4:**
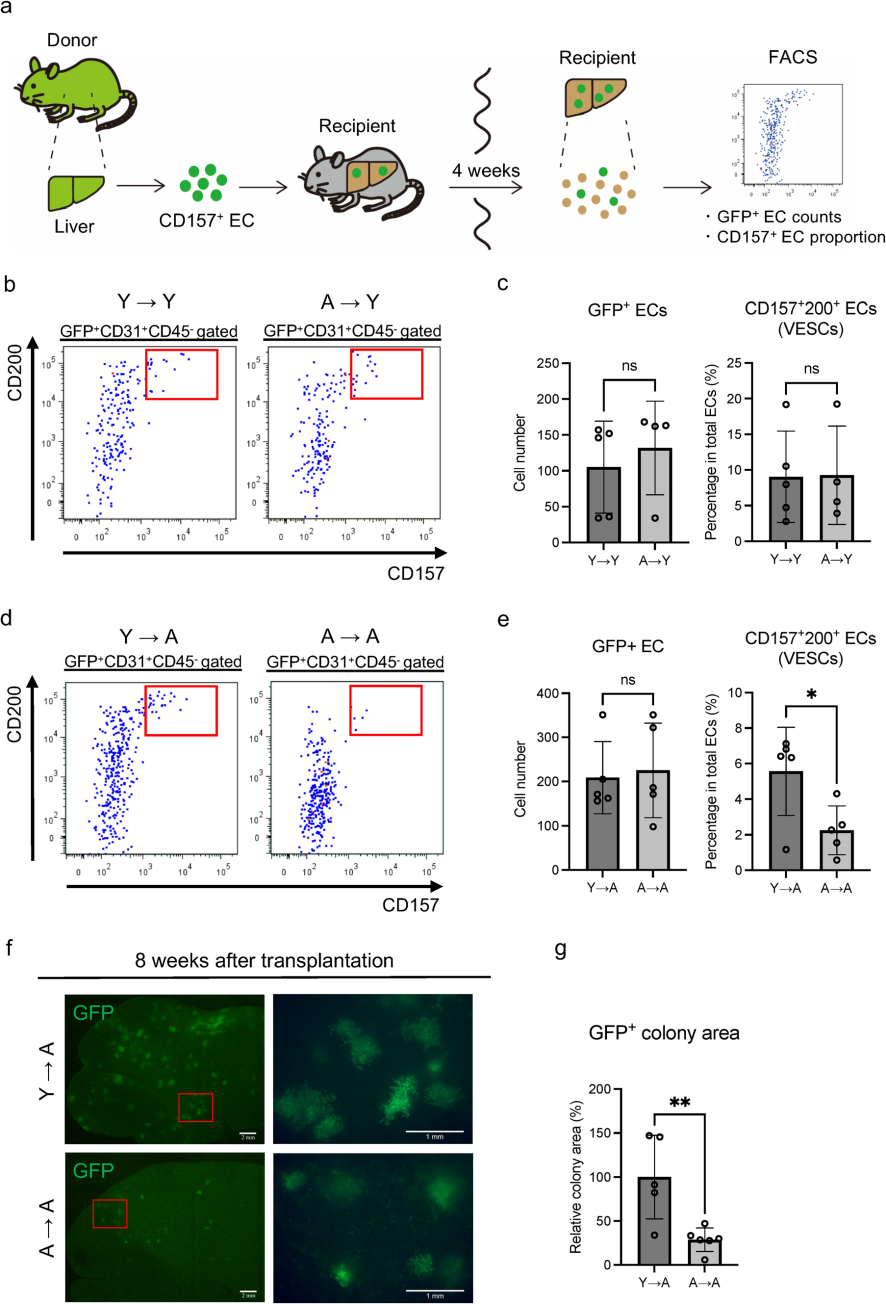
Aged VESCs and their progeny grow less efficiently than young VESCs when transplanted to aged recipients but not to young recipients. **a** Schematic diagram of VESC transplantation assays. CD157^+^CD200^+^ VESCs were isolated from EGFP mouse liver and orthotopically transplanted into recipient wild-type mice. Four weeks after transplantation, recipient livers were analyzed by FACS to evaluate the engraftment of GFP^+^ ECs and the proportions of VESCs among the engrafted cells. **b** Five thousand VESCs were isolated from the liver of young (2-3 month-old) and aged (25-26 month-old) EGFP donor mice, followed by transplantation into young (2-3 month-old) wild-type recipient mice. Representative FACS plots of engrafted ECs (GFP^+^CD31^+^CD45^-^ population) 4 weeks after transplantation are shown. Red boxes denote the VESC population. Y→Y: transplantation from a young donor to a young recipient. A→Y: transplantation from an aged donor to a young recipient. **c** Quantification of total engrafted ECs and the proportion of VESCs therein in (**b**) (*n* = 4 or 5). **d** Five thousand VESCs from young and aged EGFP donor mice were transplanted into aged (24-25 month-old) recipient mice. Representative FACS plots of engrafted ECs are shown. Y→A: transplantation from a young donor to an aged recipient. A→A: transplantation from an aged donor to an aged recipient. **e** Quantification of total engrafted ECs and the proportion of VESCs therein in (**d**) (*n* = 5). **f** Fluorescence stereoscopic images of aged recipient livers 8 weeks after transplantation of young or aged VESCs. Higher magnifications of the areas indicated by the red box in the left-hand panels are shown in the right-hand panels. Scale bars represent 2 mm (left) and 1 mm (right), respectively.  **g** Quantification of the relative colony area in (**f**) (*n* = 5 or 6). Statistical significance was assessed with two-tailed unpaired Student’s *t* tests in (**c**), (**e**) and (**g**). *P < 0.05, **P < 0.01, ns : not significant.

To evaluate the proliferation of CD157^+^ VESCs and their progeny in vivo, we transplanted liver VESCs isolated from young or aged EGFP mice into young wild-type recipients (2–3 month-old). Four weeks later, livers from recipient mice were processed for FACS analysis to evaluate the total cell counts of engrafted GFP^+^ ECs and the proportion of GFP^+^CD157^+^ VESCs therein (Fig. 4a). We expected an inferior ability of VESCs from aged mice to expand in vivo, but we found no difference in the total number of engrafted ECs as well as the CD157^+^ EC fraction 4 weeks (Fig. 4b and c) and also 12 weeks after transplantation (Fig. S3b–d). No GFP-positive cells were observed in the recipient lung, suggesting that VESCs transplanted into the liver did not home to other organs (Fig. S3a). Next, we transplanted VESCs into aged recipient mice. Four weeks thereafter, the number of engrafted CD157^+^ VESCs derived from aged donors was significantly lower than from young donors (Fig. 4d and e). However, the total cell counts of engrafted ECs isolated from aged recipients was similar for young and aged donors at this time. But 8 weeks after transplantation, the reconstituted vascular area marked by GFP was significantly smaller in mice injected with aged donor-derived ECs (Fig. 4f and g). These findings are consistent with the results of the in vitro colony-forming assays, indicating that the lesser potential of aged VESCs to produce ECs can be ameliorated in an appropriate microenvironment. In contrast, histological analysis showed that both young and aged VESCs differentiated into mature ECs residing in the sinusoidal vessels, portal veins and central veins after transplantation, irrespective of recipient age (Fig. S3e and f). This suggests that the differentiation potential of VESCs was preserved to some extent during aging. Ideally, gene expression analysis using donor-derived ECs isolated from recipient mice would be required to quantitatively assess the impact of aged microenvironments on VESCs in future studies.

### Aging-induced inflammation is a possible hallmark of the aged liver microenvironment

**Fig. 5:**
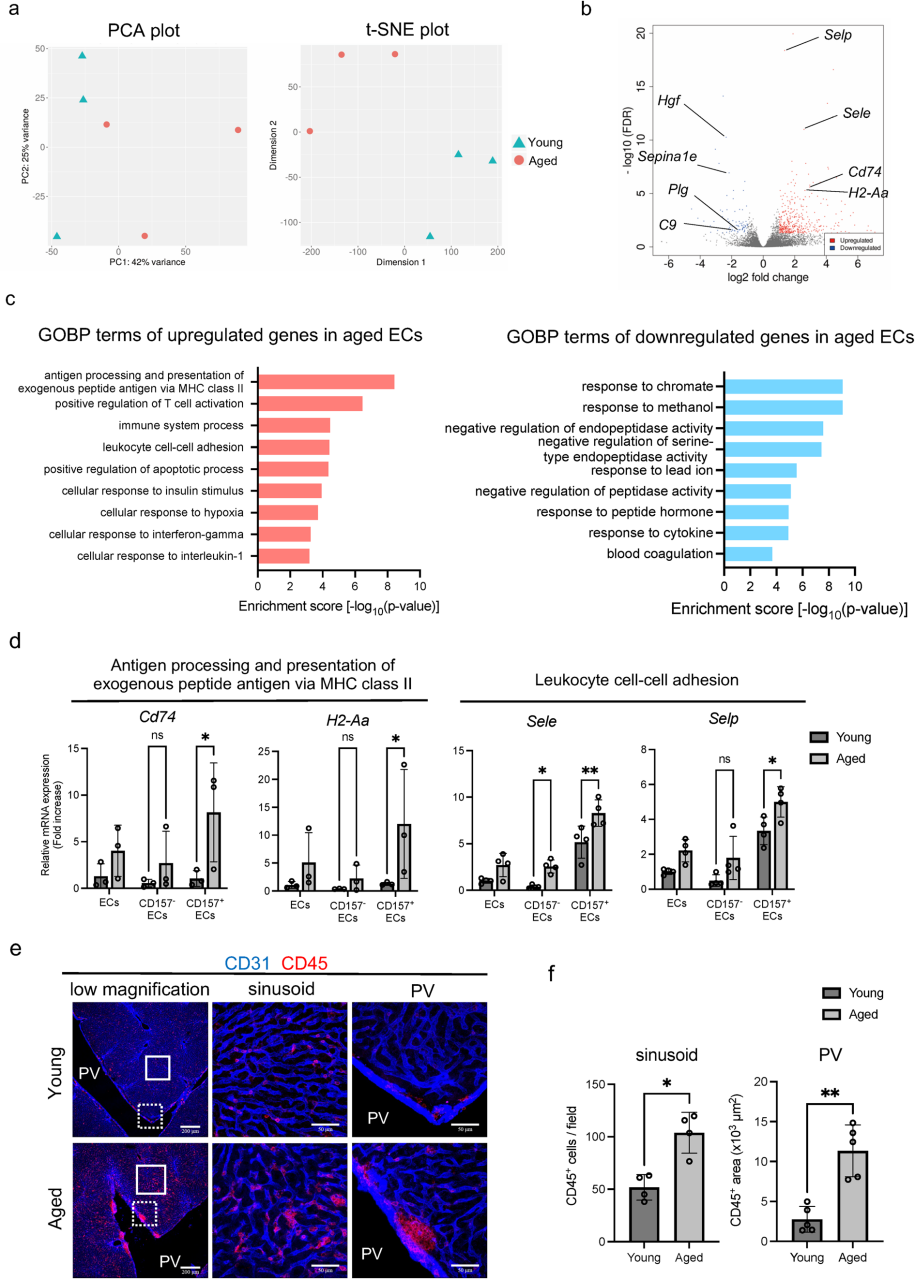
Inflammatory signaling is activated in aged ECs including VESCs. **a** Principal component analysis (PCA) and t-distributed stochastic neighbor embedding (t-SNE) plots of RNA-seq data using liver ECs (CD31^+^CD45^−^) isolated from young (10-week-old) and aged (29–30-month-old) mice. **b** Volcano plot of genes differentially expressed between young and aged liver ECs. Genes significantly up- or down-regulated (*q*-value < 0.05, log2FC > 1 or log2FC < -1) in aged liver ECs are colored in red or blue, respectively. **c** Gene Ontology (GO) analysis of up- or down-regulated genes in aged liver ECs compared to young ECs. Enrichment scores [-log_10_(p-value)] of the top nine GO biological process (GOBP) terms are shown (*n* = 3). **d** Relative mRNA levels of inflammation-induced genes in CD157^+^ and CD157^−^ liver ECs of young (3-month-old) and aged (25–27 month-old) mice quantified by qPCR. *Cd74* and *H2-Aa* belong to the GO term “antigen processing and presentation of exogenous peptide via MHC class II” and *Sele* and *Selp* to the GO term “leukocyte cell-cell adhesion”. Statistical significance was assessed with two-way ANOVA followed by Sidak’s post hoc testing. (*n* = 3 or 4) **e** Immunohistochemical staining of young and aged liver with anti-CD31 and anti-CD45 antibodies. Left-hand panels show low magnification views. Magnified views of sinusoidal and portal vein regions (surrounded by solid and dotted lines in the left panels, respectively) are shown in the middle and right-hand panels, respectively. Scale bars represent 200 μm on the left and 50 μm in the middle and on the right. **f** Quantification of CD45^+^ cells in the sinusoidal and portal vein area in (**e**) (*n* = 4 and 5, respectively). Statistical significance was assessed with two-tailed unpaired Student’s *t* test *P < 0.05, **P < 0.01, *ns*  not significant

The results of VESC transplantation assays raised the possibility that the microenvironment in aged mice might contribute to the age-related decline of VESC proliferative potential. Thus, we aimed to identify aging-related transcriptional differences in liver ECs by RNA-seq. Principal component analysis (PCA) and t-distributed stochastic neighbor embedding (t-SNE) plots illustrate global differences between transcriptomes from young and aged liver ECs (Fig. 5a). Primary component-1 (PC-1) mainly contributed to the age difference, and was enriched for genes related to the immune response (Fig. S4a). Gene ontology (GO) analysis of differentially expressed genes revealed that upregulated genes in aged liver ECs relate to the inflammatory response, including cell adhesion molecules, immunity, and several cytokines such as interferon-gamma (IFN-γ) and interleukin-1 (IL-1) (Figs. 5b, c and S4b). Gene set enrichment analysis also indicated the upregulation of inflammatory genes in aged liver ECs (Fig. S5). Several secretory proteins such as *Hgf* and *Serpina1* as well as blood coagulation factors including *Plg* and *C9* were down-regulated in aged liver ECs (Fig. 5b and c). We next investigated the expression of inflammatory genes in CD157^+^ ECs and CD157^−^ ECs by quantitative PCR (qPCR). Results showed that relative to their young counterparts, MHC class II molecules, *Cd74* and *H2-Aa*, which are induced by inflammation in response to IFN-γ [23], were significantly upregulated in aged CD157^+^ ECs but not in aged CD157^−^ ECs. Expression of mRNA for cell adhesion molecules *Sele* and *Selp*, which are downstream target genes of NF-κB upregulated by inflammation [24, 25], was increased both in CD157^+^ and CD157^−^ ECs with age (Fig. 5d). These data suggest that activation of inflammatory signals is a major transcriptional change with age in liver ECs including VESCs. Immunohistochemical staining showed that CD45^+^ immune cells are more abundant in aged liver than young liver in both sinusoidal and periportal regions (Fig. 5e and f). The Tabula Muris Consortium performed scRNA-seq on 23 organs and tissues across the lifetime of *Mus Musculus* [26, 27]. This shows strong enrichment of genes related to immune response pathways in aged mice across organs including the liver. We analyzed young (3-month-old) and aged (24-month-old) liver data from Tabula Muris Senis (Fig. S6a and b) and found that several types of immune cells increased with age while sinusoidal ECs decreased (Fig. S6c). *Bst1*-positive VESCs were not detected in this analysis probably because of the difference in the cell isolation protocol. Kupffer cells increased from 6.43% of total cells to 11.39% with age. *Cd68*-positive myeloid leukocytes (monocyte/macrophage lineage) rose from 2.33 to 12.24% and *Cd3d*-positive T cells (including mature NKT cells, CD8 T^+^ cells, CD4^+^ T cells and other T cells) increased from 4.65 to 17.3% as well (Fig. S6c and d). Gene expression analysis revealed that *Ccl4* and *Ccl5* were upregulated in *Cd3d*-positive T cells with age. *Ccl9* and *Il1b* were increased in aged Kupffer cells and *Cd68*-positive myeloid leukocytes (Fig. S6e and f). Although *Ifng* and *Tnf* were rarely expressed in immune cells in the liver, we cannot exclude the possibility that these cytokines were produced in the intestine and transported to the liver, as shown in an earlier study [22]. Another scRNA-seq study focusing on liver EC subtypes identified an inflammatory EC subtype specific for aged mice [28]. Our data are in line with these previous findings and further show that inflammatory responses are still activated at older age (up to 30 months) and possibly affect the VESC population. It has been reported that aging-induced inflammation impairs stem cell functions such as self-renewal and differentiation in several types of tissue-resident stem cells [29–31]. Thus, we propose that the inflammatory microenvironment of the aged liver might contribute to the retarded growth of VESCs in vivo.

## Discussion

In the present study, we found that there are fewer tissue-resident VESCs as well as sinusoidal ECs in the liver of older than younger mice. In vitro culture experiments revealed that the ability of VESCs from the mouse liver and lung to generate ECs is impaired with age. Orthotopic transplantation showed that aged liver VESCs and their progeny expand less efficiently than their young counterparts when transplanted into aged mice, but are equally functional in young mice. We investigated gene expression profiles by RNA-seq and qPCR, and found that the expression of inflammation-related genes including several cell adhesion molecules was enriched in aged liver ECs including VESCs. scRNA-seq analysis using the Tabula Muris Senis data revealed that immune cells, especially T cells and monocyte/macrophage lineage cells, were enriched in the aged liver. These cells produced several chemokines and IL-1b, and thus might contribute to age-associated inflammation in the liver.

Such aging-associated inflammation, so-called inflammaging, is reported to be detrimental to several types of tissue-resident stem cells including hair follicle stem cells [29], hematopoietic stem cells [30] and neural stem cells [31]. In the present study, RNA-seq and qPCR analysis showed that inflammation markedly contributes to the age-associated transcriptional changes in liver ECs including VESCs (Fig. 5a–d). We also confirmed the enrichment of inflammatory cells in the aged liver (Figs. 5e, f and S6). These findings indicate that the activation of inflammatory signals is a primary signature of aged VESCs. However, data illustrating how inflammation affects VESCs mechanistically is lacking in the present study. This limitation arises because aged mice do not survive well after monoclotalin treatment and irradiation; hence, we were unable to obtain sufficient mice to analyze. Although previous studies showed that age-associated inflammation dysregulates stemness of several types of stem cells, at this time, it remains speculative as to whether inflammation is actually the cause of the proliferative deficit of VESCs. Further investigations are needed to elucidate causal relationships.

It has been reported that there is a sex-dimorphism in the aging of the immune systems, especially in B cells and monocytes [32]. As only female mice were employed in the present study, comparison of male and female mice is required to investigate differences between the sexes with age. Because inflammatory signals are more highly activated and monocytes are relatively abundant in aged men compared to women [32], male mice might be more susceptible to the aging of VESCs.

In addition, genes associated with phagosome formation, membrane trafficking and lysosomal acidification were enriched in young liver ECs (Fig. S5a). These biological processes contribute to protein homeostasis. Because loss of protein homeostasis is regarded as a primary hallmark of aging and contributes to stem cell aging[16, 33], relative down regulation of these genes in aged ECs might also contribute to the proliferative deficits of VESCs.

Our data suggested that the ability of VESCs to generate ECs is impaired with age. However, we also showed that this impairment is reversible because generation of ECs by VESCs from aged or young mice was similar in a young microenvironment. This might be because of alterations in cell-extrinsic factors such as the stem cell niche and circulating factors [34]. Rejuvenation of aged stem cells on transplantation into a young microenvironment or by means of heterochronic blood exchange has been previously reported [35, 36]. Identification of the putative rejuvenating factors for VESCs could pave the way to establish a new strategy for anti-aging therapy, and needs further investigation.

Because the number of VESCs decreases with age, only 4,000–5,000 VESCs were available from liver of each aged mouse (Fig. 1b). To overcome the problem of the low number of VESCs in old mice, we needed to pool them from several mice. However, the viability of ECs worsened when a large amount of liver was simultaneously treated. Therefore, the availability of aged VESCs was a major technical limitation for the current study especially in the transplantation assays. If and when it becomes possible to prepare single-cell suspensions more quickly in the future, serial transplantation analysis to evaluate the long-term repopulating potential and differentiation of vascular ECs into different types such as arterial, venous and sinusoidal ECs after transplantation will be performed.

Although mice with grossly visible lesions were excluded from this study, pathological assessments were not performed. Because the aged mice used here are very old (> 24 months), it should be noted that some pathological changes might have affected the experimental outcome. Additional experiments with pathological assessments or using younger mice (18–20 months) are required to further confirm the conclusions in the present study.

The loss of VESCs with age seems to coincide with the decline of the whole EC population and the vascular density in the liver, but causal relationships remain to be elucidated. In addition, it will be desirable to evaluate the contribution of tissue-resident VESCs to regeneration and physiological turnover of organ vasculature in situ (without transplantation). Further studies using CD157-CreERT mice, such as Cre-inducible diphtheria toxin receptor (iDTR) transgenic animals and CD157-lineage tracing mice are required to answer those questions. It is also important to determine how VESC aging affects the whole vasculature and organ homeostasis. Such future work might establish a new strategy of anti-aging therapies targeting VESCs.

## Materials and methods

### Mice

C57BL/6J and C57BL/6-Tg (CAG-EGFP) female mice were purchased from Japan SLC (Shizuoka, Japan) and CLEA Japan (Tokyo, Japan). Young mice were 2–3 months-old, whereas those older than 24 months were regarded as aged. Mice with grossly visible tumors, hepatomegaly or splenomegaly were excluded from the study. Some but not all mice were assessed for body weight, liver weight, lung weight and serum ALT levels before the experiments began. For the quantification of serum ALT levels, blood was sampled from the heart and centrifuged to separate serum for ALT measurements using an ALT assay kit (#700,260, Cayman Chemical, Ann Arbor, Michigan) and a microplate reader (PowerScanHT; DS Pharma Biomedical, Osaka, Japan). All experiments were carried out following the guidelines of Osaka University Committee for animal and recombinant DNA experiments. Mice were handled and maintained according to the Osaka University guidelines for animal experimentation.

### EC isolation and cell counting by flow cytometry

Isolation of ECs from murine liver and lung was performed as previously described [37]. Briefly, the organs were chopped up with scissors. Tissue fragments were then digested with several enzymes including dispase (#17105-041, Gibco, Waltham, MA) and collagenases (#034-22363, Wako, Osaka, Japan and #LS004176, Worthington Industries, Columbus, Ohio) followed by mechanical dissociation using a bioshaker (#BR-22FH, Taitec, Saitama, Japan) to prepare single-cell suspensions. Cells were labeled with fluorescent antibodies and ECs were isolated by FACS. Fluorescent antibodies used were as follows: Purified rat anti-mouse CD16/CD32 (#553,142, BD Biosciences, Franklin Lakes, NJ), Brilliant Violet 421 rat anti-mouse CD31 (#102,423, Biolegend, San Diego, CA), FITC rat anti-mouse CD31 (#11-0451-85, Thermo Fisher Scientific, Waltham, MA), APC-Cy7 anti-mouse CD45 (#103,116, BioLegend), APC anti-mouse CD157 (#140,208, BioLegend), PE rat anti-mouse CD200 (#123,808, BioLegend). Propidium Iodide (PI) (#P4170-10MG, Sigma-Aldrich, St. Louis, MO) was used to exclude dead cells. The gating strategy for the isolation of VESCs was as described previously [37]. Cell populations were selected based on forward scatter area (FSC-A) and side scatter area (FSC-A), and cell doublets were excluded using forward scatter width (FSC-W) and side scatter width (SSC-W). PI-negative cells were analyzed for CD31 and CD45 expression. Finally, CD31^+^CD45^−^ ECs were divided into three fractions based on their expression of CD157 and CD200 antigens (CD157^+^CD200^+^, CD157^−^CD200^+^ and CD157^−^CD200^−^). Fluorescence minus one (FMO) controls were used to set the gates. We ran the samples at a low event rate (< 3,000 events/s) and used purity sorting mode (yield mask 32, purity mask 32, phase mask 0) to isolate ECs. In this setting, the sorting efficiency was usually 80–90% and the purity of sorted cells > 98%. Sorting efficiency was checked by counting the number of sorted cells with a cell counter (#WC2-100, Waken, Kyoto, Japan) for each experiment. Actual cell counts of sorted cells were calculated considering the sorting efficiency. In Fig. 1, the number of ECs counted by analyzing the whole cell suspension prepared from one liver by flow cytometry is illustrated.

### In vitro culture of CD157-positive VESCs

One day before the isolation of primary murine VESCs by FACS, OP9 feeder cells (#RCB1124, RIKEN cell bank, Ibaraki, Japan) were seeded into a 24-well plate at a concentration of 2.0 × 10^4^ cells/well. On the day of seeding the VESCs, the OP9 cells were almost confluent. CD157^+^ VESCs were sorted as described above and then seeded onto OP9 cells in the 24-well plates (1,000 cells/well for liver VESCs, 5,000 cells/well for lung VESCs). Cells were cultured in RPMI medium (#R8758-500ML, Sigma-Aldrich) supplemented with 10% fetal bovine serum (FBS) (#172012-500ML, Sigma-Aldrich) and 0.1% 2-mercaptoethanol (#21,985,023, Gibco). Cell culture plates were maintained under 5% CO_2_ in humidified air at 37℃. Ten ng/mL of VEGF165 (#100–20, PeproTech, Cranbury, NJ) was added to the culture medium every 3 days. Ten days after seeding, cells were fixed with 4% PFA in phosphate-buffered saline (PBS) for immunostaining. In Fig. 3c, VESCs were isolated from EGFP mice to visualize the process of the colony formation. EGFP fluorescence was observed every other day at the same position in the culture plate using a fluorescence microscope (Leica Microsystems, Wetzlar, Germany).

### Immunostaining of cultured ECs

After PFA fixation, immunostaining with rat anti-mouse CD31 antibody (#553370, BD Biosciences) was performed as previously described [38]. After the primary antibody, cells were incubated with biotin-conjugated anti-rat IgG antibody (Dako, Santa Clara, CA). Then avidin-biotin complexes were formed employing VECTASTAIN Elite ABC-HRP Kits (#PK-6100, Vector Laboratories, Newark, CA). Finally, EC colonies were visualized with 3,3’-diaminobenzidine (DAB) and nickel chloride (NiCl_2_). Images were captured with a Canon EOS kiss X7. Areas and diameters of EC colonies were quantified with Fiji software [39].

### Immunohistochemical staining

Tissue preparation and staining was as previously described [40]. Briefly, fixed liver specimens were embedded in OCT compound (Sakura Finetek, Torrance, CA) and frozen at -80℃. Frozen specimens were then sectioned at 40 μm thickness and washed with 0.1% Tween 20 in PBS (PBS-T). They were then incubated with blocking buffer (2% skimmed milk in PBS-T) for 1 h at room temperature. Primary antibodies were diluted with the blocking buffer at 1:200–1:400. Antibody was left on the sections overnight at 4℃. Primary antibodies used were as follows: rat anti-mouse CD31 (#553,370, BD), Armenian hamster anti-mouse CD31 (#MAB1398Z, Merck Millipore, Burlington, MA), rat anti-mouse CD157 PE (#140,204, Biolegend), rat anti-mouse CD45 PE (#12-0451-83, Biolegend). For primary antibodies that were not directly conjugated, sections were sequentially incubated with fluorophore-conjugated secondary antibodies diluted with the blocking buffer at 1:500 for 3 h at 4℃ on the next day. Second antibodies used were as follows: Alexa Fluor 488-conjugated goat anti-rat IgG (#A11006, Thermo Fisher), Alexa Fluor 647-conjugated goat anti-Armenian hamster IgG (#127-605-160, Jackson ImmunoResearch, West Grove, PA). Finally, sections were mounted with fluorescent mounting media (#S3023, Dako) and imaged using a Leica TCS SP5 confocal microscope. Images were processed with the Leica application suite (Leica Microsystems), and Adobe Photoshop CC software (Adobe Systems, San Jose, CA). For quantitative measurements of vascular area as well as CD157- and CD45-positive cells, the left lobe of the liver was sectioned at the middle. Sections were assessed at 20 μm thickness and > 3 images were acquired at periportal sinusoids or portal veins from each section. Vascular density was quantified using Angiotool software [41] for Fig. 2b. CD157- and CD45-positive areas were quantified with Fiji software [39] for Figs. 2d and 5f.

### VESC transplantation assay

Transplantation experiments were performed as described previously [14]. A genotoxic pyrrolizidine alkaloid, monocrotaline (MCT) (#C2401, Sigma-Aldrich) was administered to the recipient mice at 500 mg/kg intraperitoneally 48–72 h before transplantation. On the day of transplantation, recipient mice additionally underwent whole body irradiation with a single dose of 6 Gy. Liver VESCs were isolated as described above from young (2–3 month-old) and aged (25–26 month-old) EGFP mice. Five thousand VESCs in 4% FBS/PBS were transplanted directly into the recipient’s liver through a 27-gage needle. Four weeks (28 days) after transplantation, engrafted EGFP-positive ECs were counted by analyzing the recipient’s whole liver by flow cytometry. In Fig. 4f and S3, the recipient’s liver and lung were observed using a stereoscopic fluorescence microscope (Leica Microsystems). The area of engrafted colonies was quantified with Fiji software [39].

### RNA-sequencing

Liver ECs (CD31^+^45^−^) were isolated from young (10-week-old) and aged (30-month-old) mouse livers by FACS as described above. Three independent samples were collected from each group and mRNA was extracted from ECs using RNeasy Plus Micro Kits (#74,034, Qiagen, Hilden, Germany) according to the manufacturer’s protocol. RNA libraries were prepared using the TruSeq Sample Prep v2 kit and sequenced on a HiSeq 2500 (Illumina, San Diego, CA) in 75-base single-end mode. CASAVA 1.8.2 software (Illumina) was used for base calling. Sequenced reads were mapped to the mouse reference genome sequence (mm9) using TopHat v2.1.0. Fragments per kilobase of exon per million mapped fragments (FPKM) values were determined with Cuffnorm v.2.2.1. For PCA and t-SNE visualization, read counts were uploaded and analyzed using the integrated Differential Expression and Pathway (iDEP.96) (http://bioinformatics.sdstate.edu/idep96/) program [42]. Genes with low expression values (CPM < 0.5) were filtered out with default settings for normalization (Constant c for started log: log (x + c) = 1). To analyze Gene Ontology (GO) terms of differentially expressed genes, the web-based Database for Annotation, Visualization and Integrated Discovery (DAVID) 6.8 was used (https://david.ncifcrf.gov/) [43]. GSEA v4.3.1 Mac App was used to perform gene set enrichment analysis [44]. The lists of differentially expressed genes containing log-fold changes and p-values are shown in Supplementary Tables S2 and S3.

### Quantitative PCR

ECs (CD31^+^45^−^), CD157-positive ECs and CD157-negative ECs were isolated from young and aged livers by FACS. Total mRNA was extracted from ECs using RNeasy Plus Micro Kits (#74,034, Qiagen) according to the manufacturer’s protocol. mRNA was reverse-transcribed to cDNA using PrimeScript RT reagent Kits (#RR037A, Takara Bio, Shiga, Japan). Real-time PCR was performed with the LightCycler 96 System (Roche, Basel, Switzerland). Primers are listed in Supplementary Table S1. Threshold value was determined automatically within the exponential growth region and threshold cycle (Ct) value was defined as cycle number at which fluorescence passed the threshold. ΔCt value was determined by subtracting the Ct value of *Gapdh* from that of the target gene. ΔΔCt value was calculated by subtracting the ΔCt value of young CD31^+^45^−^ ECs from the respective ΔCt value of each cell group. 2^−ΔΔCt^ is presented as a fold-increase relative to young CD31^+^45^−^ ECs in Fig. 5d.

### Single-cell RNA-seq analysis using the Tabula Muris Senis data

The filtered h5ad file for FACS subsets was downloaded from the official Tabula Muris Senis repository (https://figshare.com/projects/Tabula_Muris_Senis/64982). Raw count data of liver were transferred into the R environment (v4.2.2). Next, we extracted 3- and 24-month samples and ran the standard Seurat (v4.3.0) pipeline with default parameters (log normalization, 15 PCs, and UMAP for dimensionality reduction). The Tabula Muris Consortium cell type designations were used for the clustering. The Seurat package was used for the analysis of differentially expressed genes and UMAP visualization.

### Statistical analysis

Statistical analysis was performed with Prism 9 software. Data are presented as mean ± standard deviation (SD). Paired data were evaluated with two-tailed unpaired Student’s *t* tests and comparison of multiple groups was performed using two-way analysis of variance (ANOVA). P < 0.05 was considered to be statistically significant. P values are indicated as *(P < 0.05), ** P < 0.01) or ***(P < 0.001).

### Supplementary Information

Below is the link to the electronic supplementary material.

Supplementary material 1 (DOCX 26100.2 kb)

Supplementary material 2 (XLSX 65.9 kb)

## Data Availability

RNA-seq data are available at the Gene Expression Omnibus (GEO) under accession number GSE233052.
