# Supplementary material for: Aging impairs the ability of vascular endothelial stem cells to generate endothelial cells in mice
Source: Angiogenesis. 2023 Aug 10;26(4):567–80. doi: 10.1007/s10456-023-09891-8 (PMC10542733; doi:10.1007/s10456-023-09891-8)
Supplement: Supplementary file 1 — Supplementary material 1 (DOCX 26100.2 kb) [file 10456_2023_9891_MOESM1_ESM.docx]

**Supplementary information**

Aging impairs the ability of vascular endothelial stem cells to generate endothelial cells in mice

Shota Shimizu^1,2^, Tomohiro Iba^1,3^, Hisamichi Naito^1,3^, Fitriana Nur Rahmawati^1^, Hirotaka Konishi^1^, Weizhen Jia^1^, Fumitaka Muramatsu^1^, Nobuyuki Takakura^1,4,5,6^

^1^ Department of signal Transduction, Research Institute for Microbial Diseases, Osaka University, Osaka, Japan

^2^ Department of Anatomy, Keio University School of Medicine, Tokyo, Japan

^3^ Department of Physiology, Kanazawa University School of Medicine, Ishikawa, Japan

^4^ World Premier Institute Immunology Frontier Research Center, Osaka University, Osaka, Japan.

^5^ Integrated Frontier Research for Medical Science Division, Institute for Open and Transdisciplinary Research Initiatives (OTRI), Osaka University, Osaka, Japan.

^6^ Center for Infectious Disease Education and Research, Osaka University, Osaka, Japan.

Address correspondence to Nobuyuki Takakura, M.D., Ph.D., Department of signal Transduction, Research Institute for Microbial Diseases, Osaka University, 3-1 Yamada-oka, Suita, Osaka 565-0871, Japan. E-mail: [ntakaku@biken.osaka-u.ac.jp](mailto:ntakaku@biken.osaka-u.ac.jp)

**Fig. S1**

**

**

**Fig. S1** Body weight, organ weights and serum ALT levels of mice at different ages. **a** Body weight, liver weight and lung weight of mice at the age of 2-3, 15-16, 21-22 and 27-28 months (n = 3). The ratio of liver weight to body weight is shown on the right. **b** Serum ALT levels of mice at the age of 2-3, 15-16, 21-22 and 27-28 months (n = 3).

**Fig. S2**


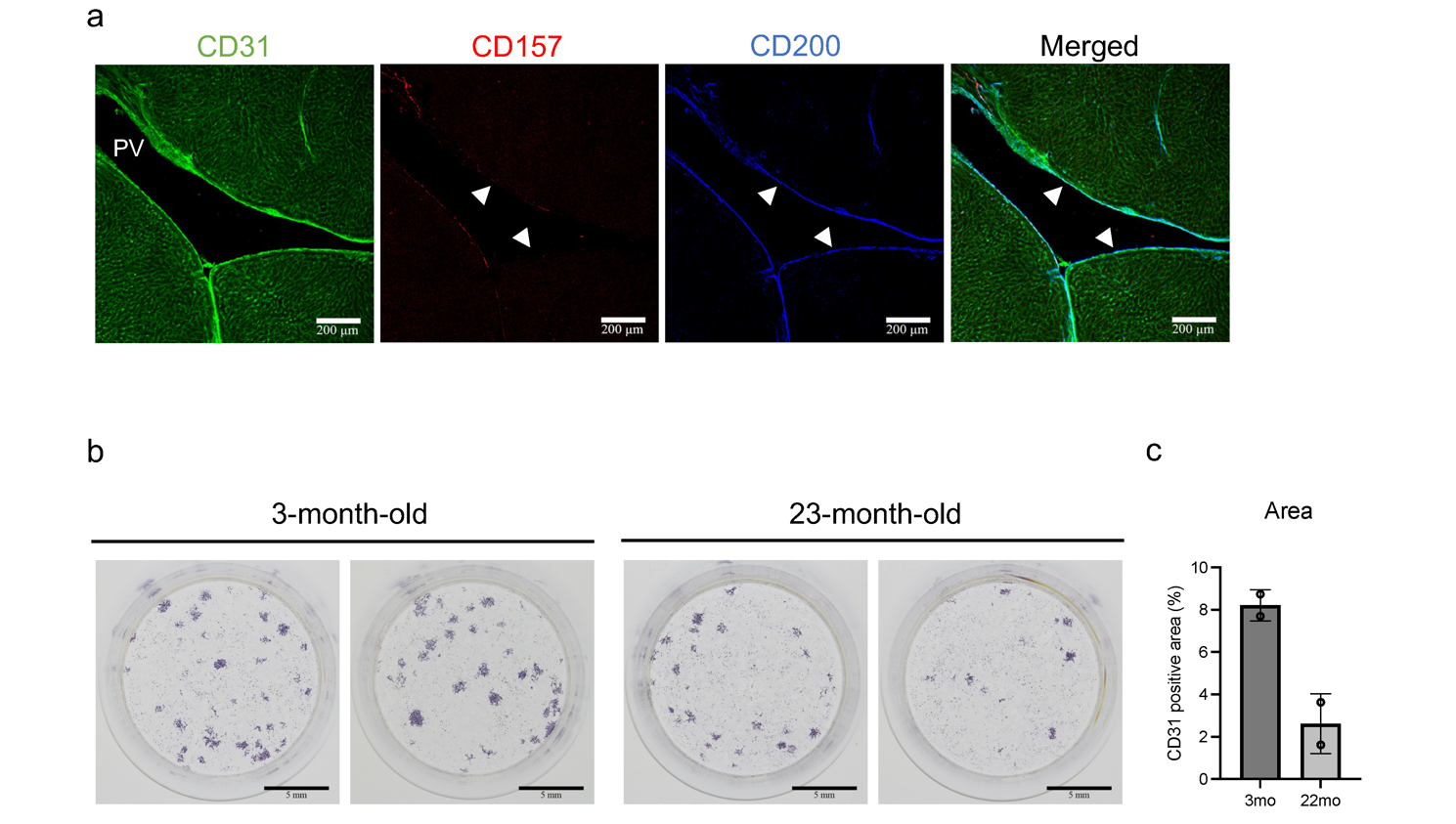


**Fig. S2** Loss of CD157-positive ECs and their proliferative potential in the aged liver. **a** Immunohistochemical staining of aged (28-month-old) livers with anti-CD31 (green), anti-CD157 (red) and anti-CD200 (blue) antibodies. Arrow heads denote CD157^-^CD200^+^ ECs in the portal vein (PV). Scale bars represent 200 μm. **b** One thousand CD157^+^CD200^+^ VESCs isolated from the livers of 3-month-old and 23-month-old mice were cultured on OP9 cells for 10 days, followed by immunostaining with anti-CD31 antibody. Scale bars represent 5 mm. **c** Quantification of CD31-positive colony area in (**b**) (n = 2).

**Fig. S3**

**
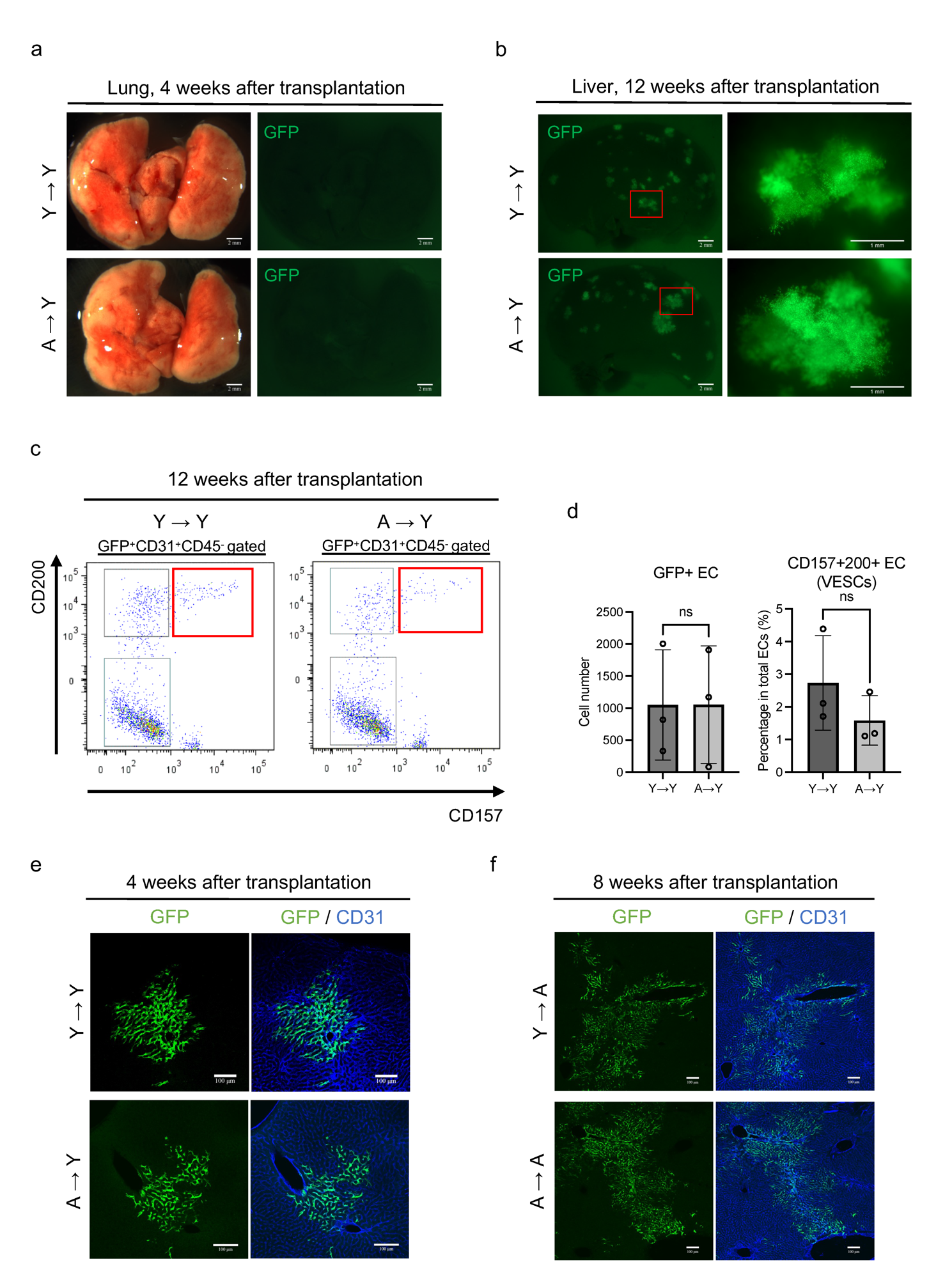
**

**Fig. S3** Transplantation of VESCs into young recipient mice. **a** Fluorescence stereoscopic images of young recipient lungs (2-3 month-old at transplantation) 4 weeks after the transplantation of young (2-3 month-old) or aged (27-28 month-old) VESCs. Each left-hand plot shows the bright-field image. Each GFP image taken at the same view is shown on the right-hand panel. Note that no GFP-positive VESCs contribute to the lung vasculature. Scale bars denote 2 mm. Y→Y: transplantation from a young donor to a young recipient. A→Y: transplantation from an aged donor to a young recipient. **b** Fluorescence stereoscopic images of young recipient livers 12 weeks after transplantation of young or aged VESCs. Higher magnifications of the areas indicated by the red box in the left-hand panels are shown in the right-hand panels. Scale bars represent 2 mm (left) and 1 mm (right). **c** Representative FACS plots of engrafted ECs (GFP^+^CD31^+^CD45^-^-gated population) 12 weeks after transplantation. Red boxes denote the CD157^+^CD200^+^ VESC population. **d** Quantification of total engrafted ECs and the proportion of CD157^+^CD200^+^ VESCs therein engrafted in (**c**). Statistical significance was assessed with two-tailed unpaired Student’s *t* test. ns = not significant. **e** Immunohistochemical staining of young recipient livers 4 weeks after the transplantation of young or aged VESCs. Livers were stained with anti-GFP (green) and anti-CD31 (blue) antibodies. Scale bars denote 100 μm. **f** Immunohistochemical staining of aged recipient livers 8 weeks after the transplantation as in (**e**). Y→A: transplantation from a young donor to an aged recipient. A→A: transplantation from an aged donor to an aged recipient.

**Fig. S4**

**
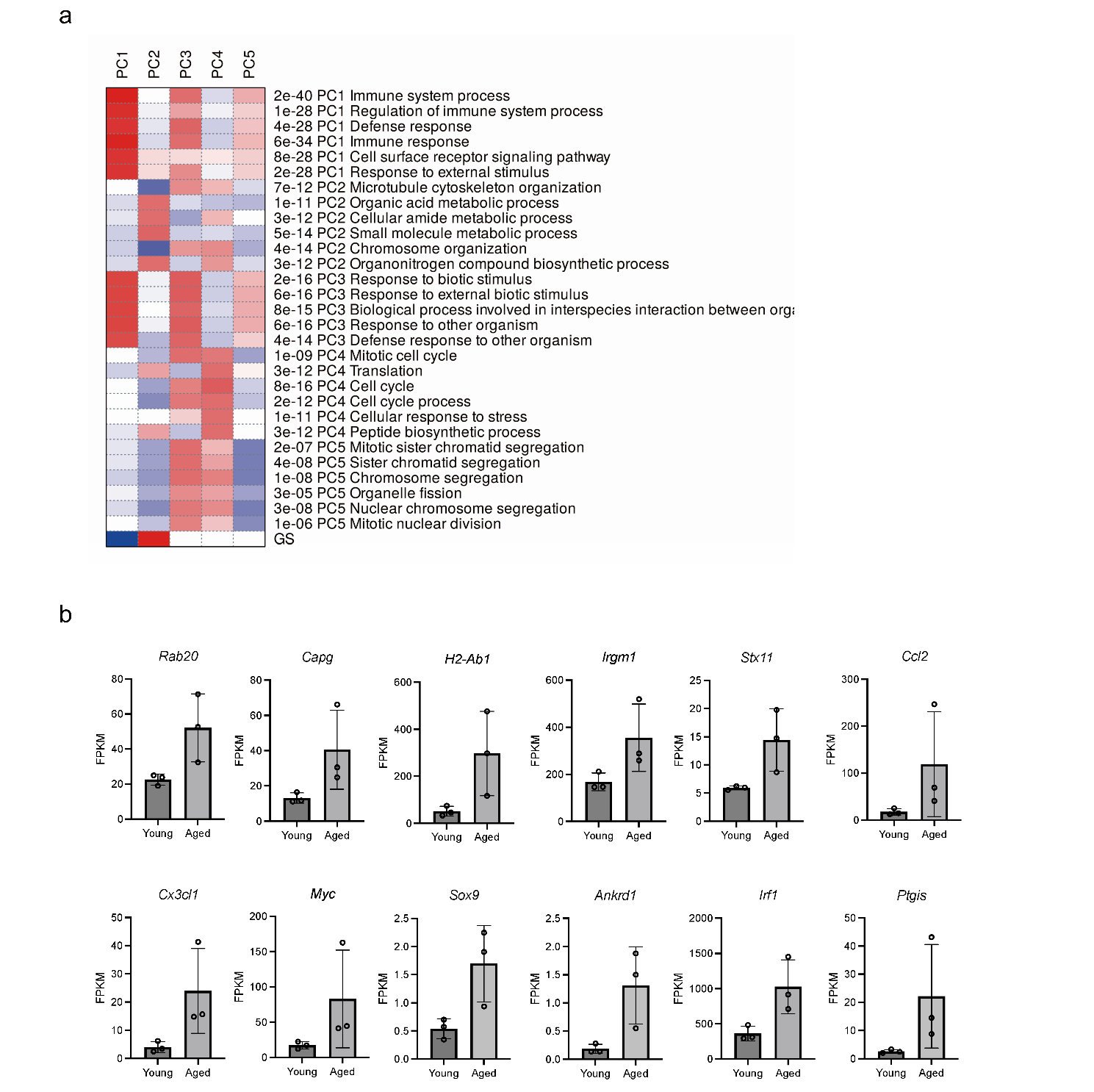
**

**Fig. S4** RNA-seq analysis of young and aged liver ECs. **a** Pathway analysis of primary component analysis (PCA) rotation. Gene ontology (GO) biological process terms and p-values related to each primary component are shown. **b** FPKM values of genes related to the GO terms “cellular response to interferon-gamma” (*Rab20*, *Capg*, *H2-Ab1*, *Irgm1*, *Stx11*, *Ccl2*, *Cx3cl1*, *Myc*) and “cellular response to interleukin-1” (*Sox9*, *Ankrd1*, *Irf1*, *Ptgis*, *Ccl2*, *Cx3cl1*, *Myc*) in RNA-seq (n = 3).

**
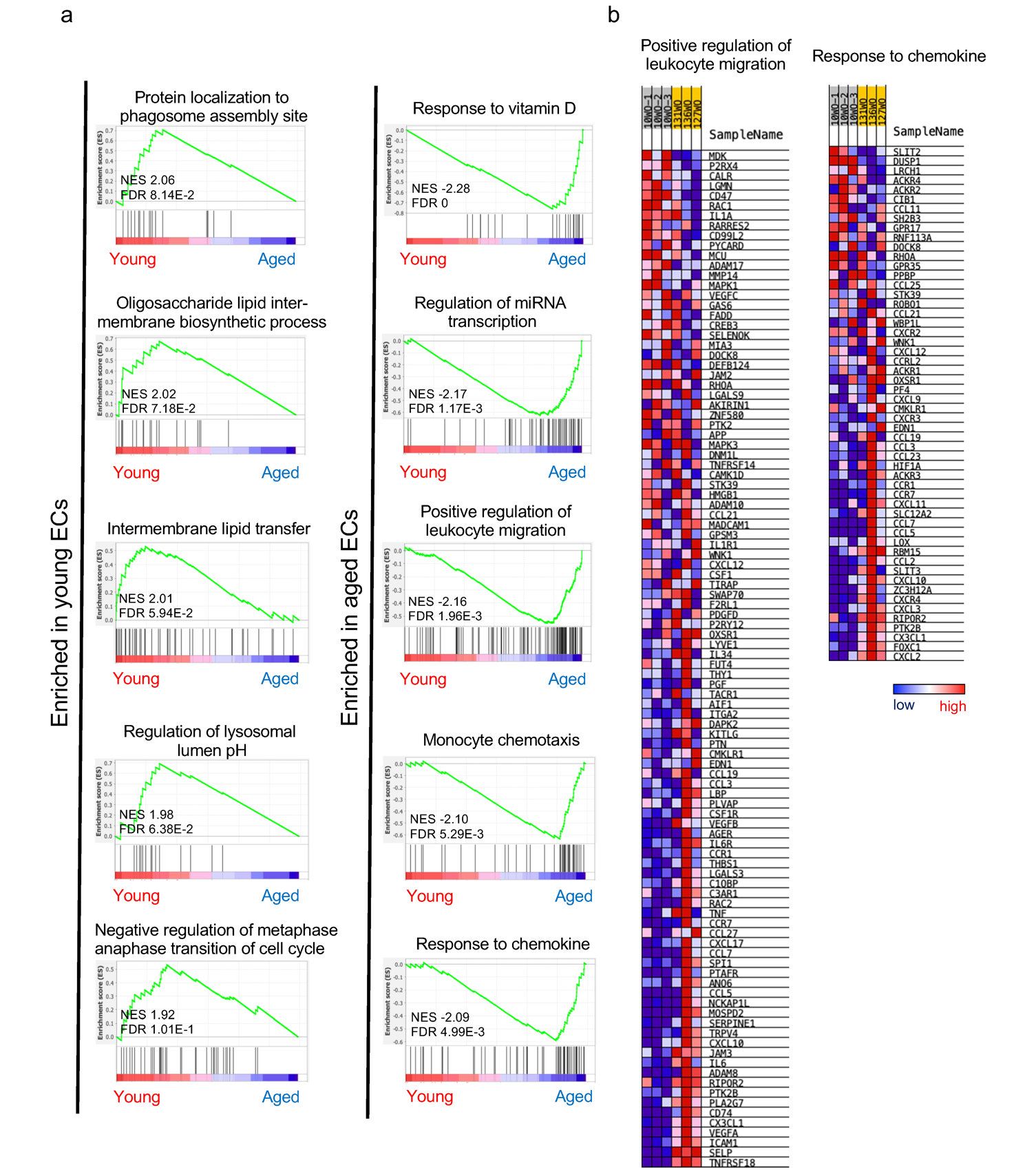
Fig. S5**

**Fig. S5** Gene set enrichment analysis of young and aged liver ECs. **a** Gene set enrichment analysis (GSEA) plots of the top 5 GO biological process terms enriched in young (10-week-old, left-hand column) and aged (29-30 month-old, right-hand column) liver ECs based on RNA-seq analysis. The top 5 GO terms for normalized enrichment scores (NES) are shown. FDR: false discovery rate. **b** Gene expression profiles of young and aged liver ECs showing constituents of the GO term “Positive regulation of leukocyte migration” (left) and “Response to chemokine” (right). The colors in the heatmaps are normalized such that the maximum value for each gene is plotted as red and the minimum value is plotted as blue.

**Fig. S6**


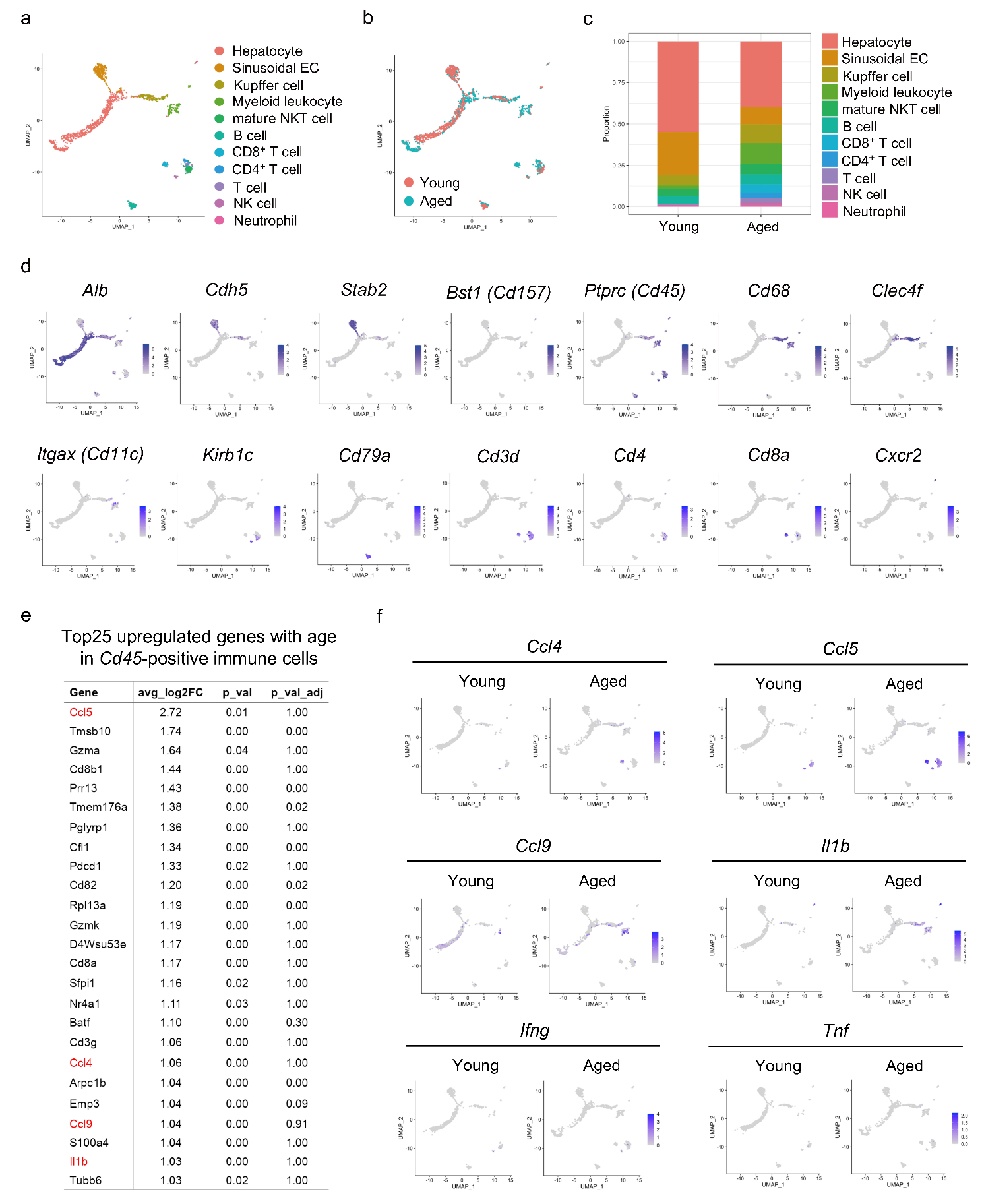


**Fig. S6**  Analysis of age-associated differences in immune cells in the murine liver using the Tabula Muris Senis data. **a** UMAP plot of young (3-month-old) and aged (24-month-old) murine liver from Tabula Muris Senis FACS data. The Tabula Muris Consortium cell type designations were used for the clustering. **b** UMAP plot in which cells were grouped by age. **c** The proportional changes of each cell type with age. **d** Expression of common cell type markers as follows: *Alb* (hepatocytes), *Cdh5* (ECs), *Stab2* (sinusoidal ECs), *Bst1* (VESCs), *Ptprc* (immune cells), *Cd68* (monocytes/macrophages), *Clec4f* (Kupffer cells), *Itgax* (dendritic cells), *Kirb1c* (NK cells), *Cd79a* (B cells), *Cd3d*, *Cd4*, *Cd8a* (T cells) and *Cxcr2* (neutrophils). **e** Top 25 up-regulated genes in aged *Cd45*-positive immune cells relative to young counterparts. Gene names of inflammatory cytokines are denoted in red. Average log2-fold changes (avg_log2FC), p-value (p_val) and adjusted p-value (p_val_adj) of each gene are shown. **f** Expression of *Ccl4*, *Ccl5*, *Ccl9*, *Il1b*, *Ifng* and *Tnf* in young and aged liver.

**Supplementary Table S1** Primers used in the present study

| Gene | Forward (5’>3’) | Reverse (5’>3’) |
| --- | --- | --- |
| Cd74 | AGATGCGGATGGCTACTCC | TCATGTTGCCGTACTTGGTAAC |
| H2-Aa | TCAGTCGCAGACGGTGTTTAT | GGGGGCTGGAATCTCAGGT |
| Sele | ATGCCTTTATGGCTGAAACC | CCAAGATTTTACAGCGAGCA |
| Selp | ACTGCCAGAATCGCTACACAG | CACCCATGTCCATGTCTTATTGT |
| Gapdh | TGGCAAAGTGGAGATTGTTGCC | AAGATGGTGATGGGCTTCCCG |
